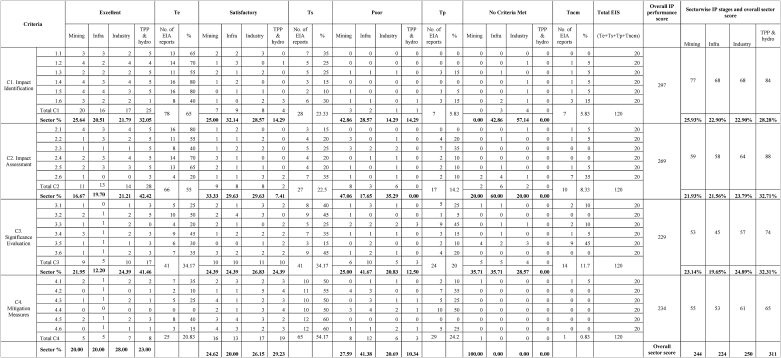# An evaluation of air quality impact prediction performance undertaken as part of environmental impact assessment (EIA) in India

**DOI:** 10.1016/j.heliyon.2024.e31263

**Published:** 2024-05-17

**Authors:** Hairul Sharani Mohd Radzuan, Jeff Martin

**Affiliations:** a University of Manchester, Humanities Bridgeford Street, Oxford Road, Manchester M13 9PL, UK

**Keywords:** Environmental impact assessment, Impact prediction, Air quality, India, Impact assessment, Megaprojects

## Abstract

Effective implementation of the Environmental Impact Assessment (EIA) is recognised as a global issue, in particular the impact prediction stage, which is the ‘core’ of EIA. Consisting of four stages: impact identification, impact assessment, significance evaluation, and mitigation measures on the possible environmental repercussions of project developmental activities, the efficacy of impact prediction can define the quality of the EIA process, which will better align environmental decision-making to sustainable development. The weakness of impact prediction in EIA demands more study to enhance practice. Although this is widely explored in the context of developed countries such as the UK, it is particularly concerning in India. A specialised review package built from several sources is utilised to assess the efficacy of air quality impact prediction, based on Lee & Colley (1991). 20 EIA reports of Category A (mega-scale projects causing significant environmental impacts) are reviewed. This study's evaluation indicates that significance evaluation and mitigation actions are the weakest phases and a major concern while assessing air quality studies conducted as a part of EIA. Recommendations to improve the process include prioritising the cumulative impact assessment within the regulatory framework, enhancing capacity building, embedding public participation and instilling accountability among stakeholders, which can be adopted globally. Additional recommendations specifically for India are revising the National Ambient Air Quality Standards (NAAQS), restructuring the EIA review mechanism by EAC and improving mitigation measures by adopting GIS and remote sensing technologies.

## 1 Introduction

Environmental Impact Assessment (EIA) is a comprehensive and proactive assessment procedure [1] that illustrates the interlinking connections between multiple scientific spheres encompassing the earth, life and social sciences [2]. The EIA process is a method for determining, anticipating, assessing and mitigating the biophysical, social, and other significant impacts of development proposals before crucial decisions are made either towards its acceptance or rejection by a governing body [3]. EIA is essential in incorporating environmental considerations into sound decision-making, influencing the future of humanity and ensuring sustainability and adherence to precautionary principles [4].

A critical component within EIA that influences its quality is the impact prediction (IP) stage, which is a crucial part of EIA reports [5]. IP applies to all the natural environmental elements such as air, water, biodiversity, noise, social, and health that are anticipated to change with the proposed project development [6,7]. Although there is long-established literature on conducting EIA and reviewing EIA reports across developed and developing nations (e.g. Refs. [[8], [9], [10], [11]], there is only a small amount of literature that specifically focuses on IP alone. Furthermore, research has highlighted the quality issues of IP presented in EIA reports, e.g. Wilton power station in the UK, Stansted airport's second runway [12], and various sectors in Romania [13].

In India particularly, this is seen in its low performance and consideration of the lack of clarity across its stages. These include process description and evaluative criteria, public involvement, assumptions, justifications of the values, transparency and accountability of stakeholders [7]. Such a problem has also been explored in recent literature that investigated issues with EIA reports that indicated the methods used to assess impacts to be biased against findings of significance. This study was done on reports in Canada, USA, Mexico, Brazil, UK, Australia and New Zealand [11]. Due to the broad nature of the problem affecting different countries, it is imperative that further studies, particularly exploring the success of EIA in developing countries are conducted.

Besides a lack of focus on a specific stage of EIA in developing countries, there is also a lack of international or locally published studies (in Indian context) specifically reviewing projects focusing on air quality impact prediction in multiple sectors, where most literature focus on a specific sector or area instead, e.g. oil and gas industry in Nigeria [14], urban project in Romania [13] and national parks in South Africa [15]. Therefore, this study intends to explore the performance of impact prediction (IP) by focusing on air quality (AQ) assessment in ensuring a high level of public health. This paper critically evaluates the four key IP stages and identifies weak stages by sampling 20 EIA reports to achieve the following objectives.

1. Create a checklist for evaluating environmental consultants by reviewing existing global guidelines and AQ in the Indian context;
2. Develop a methodology to review the performance of IP with respect to AQ;
3. Apply the best practice IP checklist on the EIA report samples;
4. Provide recommendations to help regulatory bodies and consultants improve future IP.

**Fig. 1:**
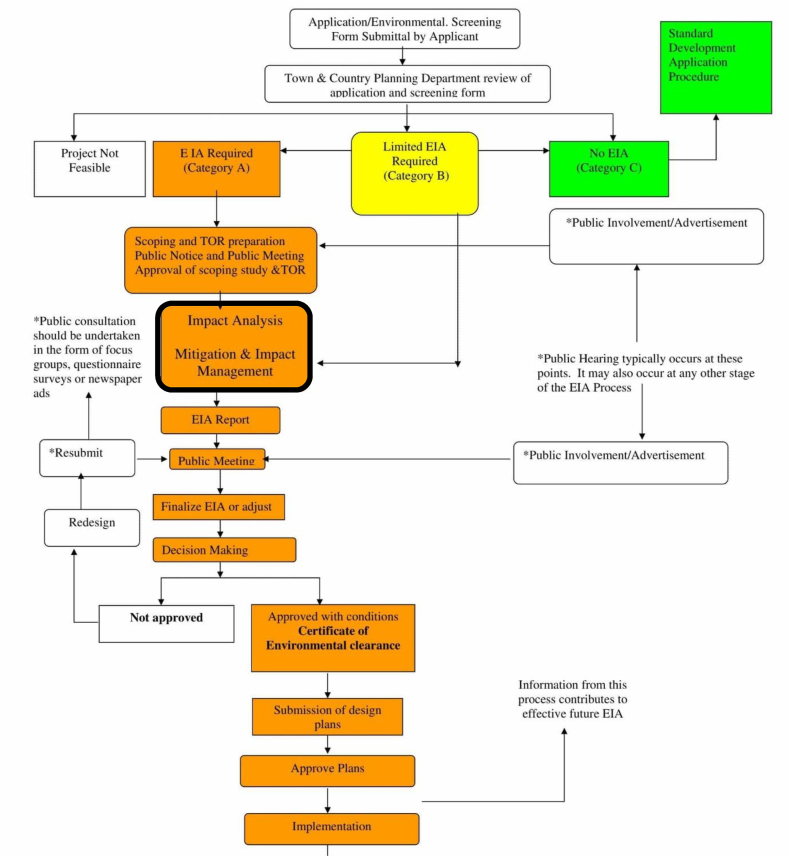
Steps involved in the Indian EIA process [17].

**Fig. 2:**
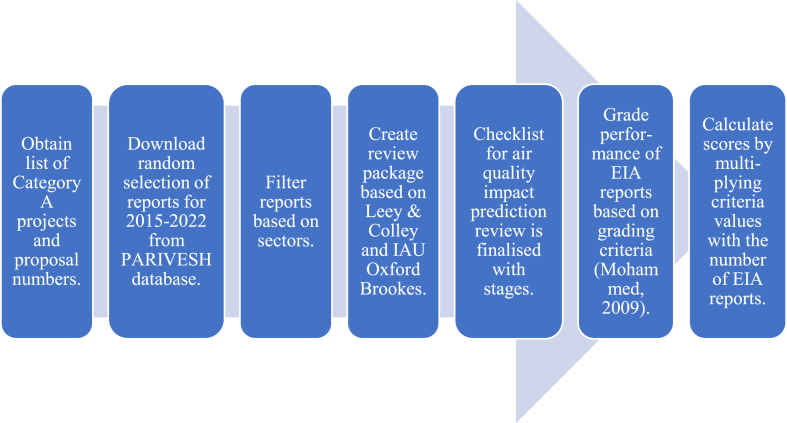
Methodological workflow.

EIA is a systematic process consisting of several steps and procedures to critically examine the environmental impacts of the intended developmental activities in advance [16] and is integrated into the administrative and procedural framework across the world. An EIA workflow implemented in India is depicted in Fig. 1(see Fig. 2).

Within the EIA process, impact prediction (IP) and analysis stages, situated around the core as shown in bold in Fig. 1, are considered critical and are referred to as the ‘heart’ of EIA [18]. This is, however, argued by other authors that precise IP is less important compared to having mitigation measures in place which can lessen negative impacts should they happen [19]. In the present study, we consider that mitigation measures are the final stage within IP and as such, are reliant on the preceding stages.

The primary objective of maintaining air quality (AQ) is to ensure a high level of public health protection [6]. AQ is characterised by the concentration and severity of air pollutants, dust and odours in a particular environment [20]. According to the data published by the World Health Organisation (WHO) (1999) [21] and Harrop (2002) [6], poor AQ is the major cause of premature death among humans and ecological health due to the extent of exposure and composition of airborne pollutants. In India, AQ remains a serious environmental health issue, and a significant number of the population resides in locations with poor AQ. Only in the early 1990s that the government implement national policy-related actions with the National Ambient Air Quality Standard (NAAQS), which was last updated in 2009 [22]. Furthermore, the National Air Quality Index (NAQI) has been recently introduced to increase public awareness of AQ management locally.

EIA was not a mandatory requirement until 1994. Only after the enactment of EIA Notification 1994 under the Environment Protection Act of 1986, EIA was recognised as a legally binding process for development projects [23]. When outlining EIA best practice principles, the IAIA and IEA (1999) [3] emphasised the importance of having a balanced approach to fit the purposes when applying the principles across the EIA process. However, several EIA studies conducted across developed and developing countries pointed to the consistent deficiency in various stages of IP in EIA reports (e.g. Refs. [8,9,13]. The weaknesses include inadequate baseline data capture, poor consideration of alternatives, and failure to effectively present and describe EIA methods and techniques [24]. Compared to developed countries, this issue is worse in developing countries, especially India [25]. Research focusing on all the IP stages in India is rare and this poses a gap in the existing literature which is Western-dominated.

This paper attempts to establish that for a comprehensive EIA study, thorough consideration and performance should be maintained across the four critical stages of impact prediction: identification, impact assessment, significance evaluation and mitigation. Furthermore, it evaluates performance across each IP stage through the quality review using best practice criteria to determine the weakest stages within the IP practice.

## 2 Methodology

This research was conducted via a desk-based study by first reviewing literature on the general EIA review in developed and developing countries from 1990 to 2023, before narrowing down to literature focusing on IP review for the same years. The data gathered for this paper were obtained from consultants who were personally approached. The study aimed to understand the relationship between EIA and IP with regard to air quality at global and national levels, with a specific focus on India. Evaluation criteria were developed based on the Lee & Colley (1991) [26] package to assess AQ practice and identify best practice frameworks, considering various types of development projects. This package originated from the UK and has been globally used in EIA reports reviews (e.g. Ref. [14] for oil and gas in Nigeria [27], for mining areas in South Africa [19], for artificial waterways in Australia and [28] for ecological sites in Sri Lanka).

Although many studies used the package to review as-is, some studies modified it to suit their application (e.g. Ref. [14]). Sample EIA reports were collected to represent different project categories from an online database, and their performance was evaluated using the developed criteria. The IP performance analysis involved examining data from the review package to determine the fulfilment of criteria across IP stages. This paper then provides suggestions on improving future IP practice in EIA, specifically for India, aiming to enhance the overall quality and effectiveness.

### 2.1 EIA reports selection criteria

A random selection of EIA reports was obtained from the EIA database maintained by the Ministry of Environment and Forests (MoEF, Government of India), known as the PARIVESH portal. To collect the Indian EIA reports for assessment, an EIA practitioner collaborated with MoEF personnel to obtain a list of Category A projects and their proposal numbers. Using these numbers, random project reports were downloaded from the PARIVESH database that represents various project sectors.

To understand the general practice across India, Category A development projects were considered, which are mega-scale projects that cause the most significant environmental impacts [29]. The environmental clearance for Category A projects is granted by 10.13039/501100001421MoEF, with duties discharged to State Pollution Control Boards (SPCB) before the public hearing [30].

EIA reports from 2015 to 2022 were selected to represent different sectors that were most relevant to current legislation. According to EIA Notification (2006), sectors under Category A are classified into four: industrial, infrastructure, mining (coal and non-coal) and power generation (thermal and hydro) [29]. From 2015 to 2022, it was found that 3431 EIA reports for Category A projects were produced within these timeframes and received environmental clearance [31]. Among these, 45 % of the EIA assessments were executed within the industry sector, followed by the infrastructure sector contributing 32 %. The rest of the EIA assessments comprised mining (coal and non-coal) and power generation by thermal/hydro.

An equal sample size was chosen from the four sectors for evaluation. Five reports were randomly chosen from each sector, assuming that each project would have a major direct or indirect impact on the AQ. The quality evaluation of 20 reports is aligned with other similar studies such as [13,14,27] which used a similar number or less. Although the number is rather small, the result can be generalised as this study analyses various sectors, the reports selected are for the most recent projects and the trends observed are compared across sectors with other international reviews. The list of EIA reports is shown in Appendix A.

### 2.2 Evaluation criteria checklist

[32] emphasised the EIA review package as a valuable instrument for evaluating quality to compare standards across sectors and periods in an unbiased and repeatable manner. A variety of review packages are available to assist stakeholders in conducting a structured and methodical evaluation of EIA reports. Several examples of the most prominent methods are the Lee and Colley package and the IAU Oxford Brookes University review package [16,33]. Since this paper focuses on AQ-specific environment components, developing evaluative criteria compatible with reviewing EIA reports of various sectors is essential.

The original and upgraded Lee and Colley package, renowned for its dependability and robustness, forms the foundation of the review methodology created [26,34]. The review package also adopts the IAU Oxford Brookes University EIS package that has evolved from the Lee and Colley method [16]. These methods have been widely adopted in EIA studies in various aspects such as [14] in Nigeria [35], in South Africa, and [36] in Bangladesh. Due to the robustness of both packages, they form the basis of review in this work which is then structured as an evaluation criteria checklist described in Table 1. Government guidance documents (MoEF) form the basis for most of the sections and criteria gathered in Table 1, combined with criteria adopted from best practices of EIA (the rest of the references), which in this case yield six criteria integrated into each section.

**Table 1:** Best practice Evaluative Checklist for AQIP study in India [16,22,[37], [38], [39], [40], [41], [42], [43], [44]].

Impact Prediction Stage	Evaluation Criteria		
1. Impact Identification	Providing information on the complete identification of sources and receptors about the development with respect to the AQ environment		
	1.1	Description of sources (line, point, area) due to processes/activities causing air pollution under varying operational conditions or phases of the development such as construction, operation and decommissioning.	
	1.2	Identification of current baseline ambient AQ through any of the below means for one season either pre- or post-monsoon: on-site measurement using samplers or analysers, a database maintained around adjacent developments (if any), the State Pollution Control Board (SPCB) or educational institutions.	
	1.3	Determination of future baseline ambient AQ using dispersion and receptor modelling software with GIS mapping, ISCST-3 (Uses Gaussian plume model) or any other approved method with a brief description of the rationales for using the method covering construction, operational and decommissioning phases.	
	1.4	Identification and quantification of pollutants with correct units as per the NAAQS Notification, (2009) during various phases of development, whichever applies to project type such as: - Sulphur dioxide (SO_2_) - Nitrogen Dioxide (NO_2_) - Particulate Matter (PM_10_ and PM_2.5_) - Ozone (O_3_) - Lead (Pb) - Carbon monoxide (CO) - Ammonia (NH_3_) - Benzene (C_6_H_6_) - Arsenic (As) - Nickel (Ni)	
	1.5	Identification of essential meteorological data from the nearest India Meteorological Department (IMD) station such as wind speed and direction, ambient air temperature, relative humidity, rainfall, atmospheric pressure and mixing height as applicable.	
	1.6	Rationale description on the selection of sampling locations for any on-site measurements in compliance with BIS/CPCB or other global competent guidelines and norms.	
2. Impact Assessment	Analysis of collected data to determine the magnitude, spatial extent, probabilities and ambiguities of the impacts.		
	2.1	Comprehensive comparisons of the baseline ambient AQ level with Indian AQ standards as set by NAAQS (2009)	
	2.2	Description of assessment methodology used such as qualitative or quantitative type for assessing the AQ impacts. Examples include numerical dispersion, analysers, the Gaussian model, ARAI Emission inventory and predictive method developed in association with AQ experts.	
	2.3	Aptness, clarity and justification of the data inputs used for the estimation of magnitude in line with the type and scale of development and associated dispersion complexities.	
	2.4	Mention of national, regional or local database sources such as: - National Ambient Air Quality Standard (NAAQS) - Central or State Pollution Control Board (CPCB/SPCB) - State or District Environmental Impact Assessment departments (SEIAA/DEIAA)	
	2.5	Future impacts forecasted output is in the form of a comparable value with the appropriate unit using either the qualitative Chemical Mass Balance (CMB) method or warranted quantified dispersion modelling methods.	
	2.6	Mention or explanation of areas that faced uncertainties while carrying out assessments or constraints in information collection.	
3. Significance Evaluation	The geographical extent and degree of variation from the current ambient AQ due to the determined magnitude of AQ impacts on proximate receptors		
	3.1		Brief description of instances when impact sensitivity could exceed the threshold on affected proximate receptors in both construction and operational phases.
	3.2		Inclusion of emergency, accident and standby protocols at the time of exceedance above threshold limits and selection of alternatives. (Additional score if scenario of with and without project discussed)
	3.3		Defining significance criteria such as reversibility, irreversibility, long or short-term impacts, proximity to sensitive areas and whether the emphasis is given to higher significant impacts than lower significant ones and justifications provided for this difference.
	3.4		Clarity of the factors evaluating significance and description of tools using either checklists, mapping or matrices. Thereby ensures the monitoring of AQ parameters based on location, reporting schedules, frequency and method of sampling evaluation during both construction and operational phases of development as briefed in the Environmental Management Plan (EMP).
	3.5		Description of cumulation AQ impact of all sources of emissions on the ambient AQ and level of public participation.
	3.6		Level of consideration to relevant primary and secondary legislations such as Air Act (1981), Environment Protection Act (1986) and Fly Ash Notification (2009) wherever applicable and methodological compliance with EIA guidance manuals for the appropriate project types such as mining, building construction and area development projects (infrastructure), thermal power plant and specialised industries.
4. Mitigation Measures	Air pollution regulating measures were implemented to reduce the project's AQ impacts across the construction and operational phases.		
	4.1		Importance or priority was given towards the reduction of AQ impacts at the source.
	4.2		Fulfilment of standard mitigation practices proposed within the EIA guidance manuals appropriate to the development type. Additionally, examine if the proposed mitigation measure is reliable, applicable and clearly defined concerning the scale and nature of the project proposed in the construction, operational and decommissioning phases.
	4.3		A brief description that outlines the performance review of mitigation measures post-decision-making through periodic or scheduled environmental audits.
	4.4		Addressing the effectiveness of mitigation measures with the identification of gaps. Additionally, are details of criteria and indicators mentioned based on which monitoring is executed?
	4.5		Description of pollution control devices towards managing air and fugitive dust emissions within and around the site. Additionally, disclosure through the attachment of Air Pollution emission permits obtained from CPCB/SPCB in case of existing development intending to expand.
	4.6		Proposal of monitoring arrangements where uncertainty over the impacts exists with details such as its functions, costs and timelines for its installations.

As the criteria are collated from government guidance for EIA and best practices as applied in previous work, the framework is considered complete as they are consolidated from real EIA reports. Based on research, there is no specific number of sub-categories used in the checklist to form the evaluation criteria, so using six for each section ensures reliability, adaptability and comprehensiveness in conducting report review.

### 2.3 Performance grading criteria

The performance grading criteria are derived from Ref. [45] with each criterion developed and evaluated on the extent of their fulfilment. In this present study, a team of two researchers who are sufficiently familiar with the requirements of EIA assessed the report performance. Working independently, the assessment is recorded in a collation sheet using the score shown in Table 2. Acknowledging the subjectivity of assessment and to avoid bias, we compared the differences in the results, re-examined and discussed the differences. Once consensus is reached, the final scores are recorded and presented in this paper. A similar approach with some modifications to the Lee and Colley package has been applied in Refs. [[46], [47], [48]].

**Table 2:** Grading of the criteria.

Performance Grade/Score	Criteria Quality Standards
3	Excellent criteria performance with no tasks left incomplete
2	Satisfactory criteria performance despite omissions
1	Poor criteria performance with many inadequacies
0	No criteria met or criteria completely overlooked

Once the criteria are evaluated, the values are multiplied by the number of EIA reports for each sector, providing a weighting between the IP stages and project sectors. Complete scores are illustrated in Appendix B.

## 3 Results

This section presents the evaluation results based on the performance against the developed air quality IP checklist. Section 4.1 outlines the IP performance between the four project sectors; section 4.2 presents the overall IP performances across different stages, and section 4.3 discusses grade valuation across IP stages. The detailed results and associated calculations are shown in Appendix B.

### 3.1 Impact prediction (IP) performances between the four project sectors

#### 3.1.1 Impact identification

For the Impact Identification stage, thermal and hydro projects performed the best with a score of 84, followed by mining with a score of 77. The sectors with the lowest scores are infrastructure and manufacturing, with an identical score of 68.

#### 3.1.2 Impact assessment

In the impact assessment stage, thermal and hydro projects performed the best with a score of 88, surpassing the manufacturing, mining and infrastructure sectors with scores of 64, 59 and 58, respectively.

#### 3.1.3 Significance evaluation

The thermal and hydro projects also lead in the significance evaluation stage with a score of 74, followed by manufacturing industries with a score of 57 and mining with 53. The infrastructure sector is at the bottom with a score of 45.

#### 3.1.4 Mitigation measures

Regarding the implementation of mitigation measures across sectors, the thermal and hydro projects still lead with a score of 65, followed by a marginal difference of 4 for the manufacturing industries at 61. The lower tier has the mining and infrastructure sectors at 55 and 53.

### 3.2 Comparison of IP performance among project sectors

**Fig. 3:**
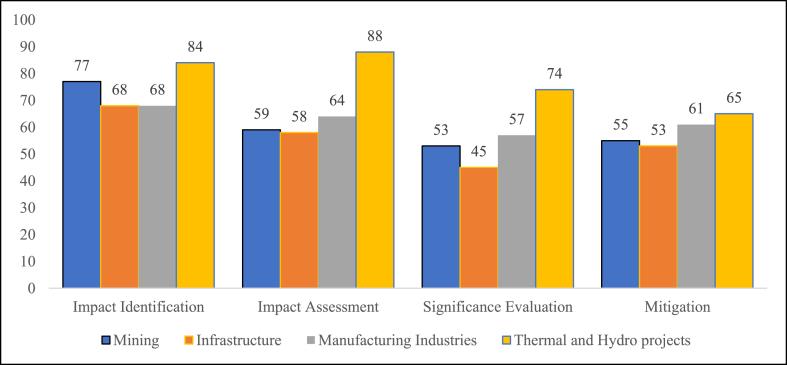
Overall comparison of IP performance across sectors.

Fig. 3 demonstrates the overall comparison of IP performance across sectors. A downward trend can be seen in the performance as a project progresses through different stages of IP, except for thermal and hydro where it improved towards the end. The impact identification stage performance is better than other stages across the project sectors, with the exception only of thermal and hydro projects for which the impact assessment stage scores the highest. Significance evaluation, which is closely followed by mitigation measures, is seen to be the weak performing stage in IP across all sectors.

The thermal and hydro sector conducted the significance evaluation and mitigation measures stage better than others, but its practice and performance are poorer. Subsequently, the infrastructure sector performed the worst in the significance evaluation stage and was almost on par with the mining sector's score in the mitigation stage. A similar trend is also apparent in the manufacturing industry sector.

For the overall total score achieved towards IP performance, the thermal and hydropower sector is leading with a score of 311. The second-best performing sector is the manufacturing industry, followed by mining with scores of 250 and 244. The lowest-performing sector is infrastructure with a score of 224.

### 3.3 Overall IP performances across different stages

Based on the evaluation checklist, the overall performance of IP reveals that the impact identification stage is the most comprehensive (score 297), followed by the impact assessment (score 269). The performance of mitigation measures is only marginally better (234) than the significance evaluation by 5 points (229). However, as this is very fine, the significance evaluation and mitigation measures can be deemed as the weakest due to the large difference.

### 3.4 Grade valuation across IP stages

Across all IP stages and project sectors based on the grade valuation categories in Table 2 and the fulfilment of checklist criteria in Table 1, the projects are categorised into percentages of excellent, satisfactory, poor and no criteria met. Table 3 highlights the summary of the analysis, while the full detailed results are supplied in Appendix B for further reference.

**Table 3:** Percentage of the fulfilment of best practice IP checklist criteria.

Parameter	Excellent (%)	Satisfactory (%)	Poor (%)	No criteria met (%)
Impact Identification	65.00	23.33	5.83	5.83
Impact Assessment	55.00	22.50	14.17	8.33
Significance Evaluation	34.17	34.17	20.00	11.67
Mitigation Measures	20.83	54.17	24.17	0.83

Out of the 20 EIA reports, 65 % of them are excellent in terms of impact identification, followed by 23.33 % providing satisfactory results and 5.83 % for both with poor fulfilment and no criteria being met. For the fulfilment of impact assessment criteria, 55 % of the EIA reports were excellent, 22.50 % were satisfactory, 14.17 % were poor, and 8.33 % did not fulfil the required criteria.

The poor fulfilment and no criteria met columns of the significance evaluation stages as highlighted show an increasing percentage of 20 % and 11.67 %, respectively. The same is true for the case of mitigation measures, where 24.17 % of the reports had poorly fulfilled the required criteria.

**Fig. 4:**
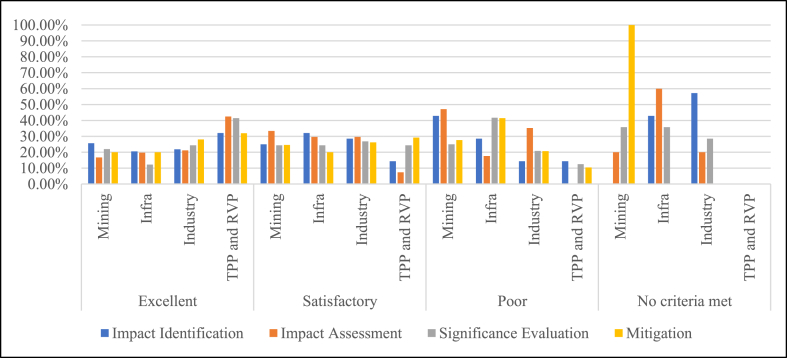
Percentage of IP valuation according to the sector.

The studied EIA reports were then categorised into criteria fulfilment based on their sectors across IP stages. They were classed into excellent, satisfactory, poor and no criteria met. It can be seen in Fig. 4 that the thermal and hydropower project sector outperformed other sectors throughout the whole EIA workflow, from impact identification to mitigation.

For impact identification performance (blue bar), the infrastructure sector (32.14 %) leads the satisfactory category, followed by industries (28.57 %), mining (25 %) and the thermal and hydropower sector (TPP and RVP) (14.29 %). The trend of the impact assessment stage (orange bar) in the satisfactory category sees the mining sector (33.33 %) leading, followed by infrastructure and industries scoring evenly (29.63 %), then thermal and hydropower projects (7.41 %). Meanwhile, 26.83 % of EIA reports within the significance evaluation stage (grey bar) are led by the industrial sector, followed by a steady score of 24.39 % by the other sectors. And finally, the mitigation measures within the satisfactory category are best fulfilled by thermal and hydropower projects (29.23 %), followed by industry (26.15 %), mining (24.62 %) and infrastructure sectors, respectively.

The mining sector is seen to score the highest percentage of the poor fulfilment of impact identification and impact assessment stages, with 42.86 % and 47.06 %, respectively, in the poor performance category. Concerning the significance evaluation and mitigation measure (yellow bar) stages, the infrastructure sector is the primary sector contributing to the poor performance with a value of 41.67 % and 41.38 %, respectively, over all the other project sectors.

The manufacturing industry sector scored the highest percentage of not meeting any criteria within the impact identification stage (blue bar), with a value of 57.14 % of the total reports. The sector with the most no criteria being met in the impact assessment stage (orange bar) is found to be the infrastructure sector (60 %). For the significance evaluation stage (grey bar), the mining and infrastructure sectors were the most that did not meet any criteria with an identical score of 35.71 % each. Under the mitigation measure stage (yellow bar), only the mining sector contributes toward not meeting a criterion. As most of the thermal and hydropower sector reports scored excellent across all IP stages, no report was found in the no criteria met in any IP stage.

## 4 Discussion and recommendations

Following the analysis of the EIA reports from four sectors of mega projects, this section discusses the strengths and weaknesses of Impact Prediction (IP) in India, focusing on the evaluative AQIP checklist. It elaborates on the performance of each IP stage across different criteria and identifies the weakest criteria in poorly executed IP stages. Afterwards, this paper provides several suggestions to enhance prediction precision, including by improving the training of environmental consultants and adhering to the latest best practice guidelines provided by regulatory bodies.

### 4.1 Performance of the impact identification stage

Generally, the impact identification stage is considered to be the least problematic within the impact prediction process as observed in England and Wales [49] and India [7]. The results in this paper support this claim, where the 10.13039/100006162EIA reports demonstrate a strong effort in identifying impacts across various project sectors. This is expected since the selected EIA reports had to describe the AQ impact as a prerequisite for further stages of the study. The reports provide detailed information on the sources, types and quantities of gaseous and particulate emissions during the different phases of the project. They also consider impacts on other conditions as guided by the published sector-wise guidance manuals. These variables are also observed in Ref. [28] who conducted an ecological impact assessment in Sri Lanka and [35] on the utility industry in South Africa.

Overall, the strengths observed in the impact identification stage include adherence to the NAAQS Notification 2009 [22] for identifying and quantifying pollutants, as well as the inclusion of essential meteorological data from the nearest IMD station and its comparison with real-time sampling values. In mining, infrastructure and industry sectors, some reports demonstrate satisfactory performance in this stage by focusing on operational impacts and disregarding non-operational conditions. However, there are several areas of improvement identified, including the need for clearer rationale in selecting baseline AQ sampling locations, providing details of the instruments used, including baseline wind speed and direction in mining projects and ensuring an adequate number of sampling locations based on sector requirements. Despite these minor shortcomings, the overall impact identification stage showed satisfactory compliance with the majority of the checklist criteria.

### 4.2 Performance of the impact assessment stage

The impact assessment stage involves analysing the magnitude of impact, spatial extent, probabilities and ambiguities [16]. Therefore, the methods practised in AQIP must be well-described. IP can involve either qualitative or quantitative analysis. For elements such as air, capturing the quantitative data helps understand the extent of environmental and human impacts [50]. In line with the global and Indian best practices, some of the numerically based air dispersion prediction models are ISCST-3, CALINE, and AEROMOD [6,51]. As no one model fits it all [6], a combination of these modelling tools can be applied for enhanced and comprehensive assessments.

According to MoEF's sector-wise guidelines (2010) [41], the current AQIP is observed to facilitate the quantitative type. In this study, most of the EIA reports used quantitative methods, except for several mining sector reports that were qualitative. Nonetheless, 7 EIA reports did not perform well in describing the method used for the modelling. The reports that came from the mining, infrastructure and industry sectors were the lowest performing. It was seen that almost 85 % of the EIA reports satisfactorily mentioned and compared the values against national standards (NAAQS) with the description of regional or local database sources maintained by CPCB/SPCB or SEIAA/DEIAA. It was also seen in a study by Ref. [52] where the method used was not clearly outlined in the report or described against national standards in Bangladesh.

The justifications in areas that faced uncertainties while conducting assessments and, in some cases, the constraints encountered during information collection are essential if further checks are required in the EIA process. This is necessary as EIA is intended to complement the decision-making process and, influence stakeholders' involvement and final judgement [53,54]. This was a low-performing criterion in this stage where almost 45 % of the reports were categorised as poor with no mention of any uncertainties or constraints faced. The description and assessment of environmental impacts are crucial when deciding the mitigation measures to be implemented [1].

Overall, the impact assessment stage scored second, following the impact identification. The thermal and hydropower projects (32.71 %) were the best-performing sector, followed by manufacturing industries (23.79 %), mining (21.93 %) and infrastructure (21.56 %) shown in Appendix B.

### 4.3 Performance of the significance evaluation stage

The significance evaluation stage involves interpreting the magnitude of impacts and their adverse effects on the environment, accounting for factors such as intensity, duration, certainty and the characteristics of the local community and politics, making it crucial. This stage is subjective and relies on the specific context and sensitivity of the environment. To ensure transparency and capture relevant information, open communication and accountability among the environmental consultant, project proponent, and regulatory bodies are needed. In India, the decision-making responsibility lies with the Expert Appraisal Committee (EAC) appointed by the Ministry of Environment, Forest and Climate Change (MoEF) for Category A projects. It is the consultant's responsibility to prepare the EIA report following the sector-specific guidance and complying with relevant legislation.

Based on the EIA reports evaluated, the significance evaluation stage overall performed the weakest. Some of the requirements that have been completed satisfactorily were descriptions of instances when AQ impact sensitivity could exceed the threshold on affected proximate receptors during construction and operation. Most EIA reports also provided appropriate emergency, accident and standby protocols at the time of air emission exceeding the threshold. They evaluated significance and described qualitatively using checklists or matrices followed by an appropriate Environmental Management Plan (EMP) for each project. In terms of adherence to the MoEF guidance manuals, 16 EIA reports performed satisfactorily. In other developing nations such as Sri Lanka, it was reported that evaluations used out-of-date information and the data used out of context, making the report contents inaccurate [28].

The weakest criteria in the study were the lack of discussion on cumulative impact assessment and poor public participation, accounting for 55 % of the identified weaknesses. Among all the project sectors, only thermal and hydropower projects showed satisfactory execution. The mining, infrastructure and industry sectors were identified as the worst, where the mining sector completely disregarded cumulative impact assessment. Although the industry and infrastructure sectors attempted to address this gap, their performance was poor, e.g., the infrastructure sector briefly mentioned traffic increase as an impact without further investigation or justification, neglecting available traffic emission data. The evaluation of IP also lacked proper description and information on receptors’ proximity and sensitivity (11 EIA reports). Generic statements without specifying the actual location and distance from the site boundary were frequently used, hindering the understanding of whether emissions reached specific sensitive receptors or not.

In India, the thermal and hydro powerplant sector (32.31 %) comparatively has a better practice in the significance evaluation stage, followed by the industry (24.89 %), mining (23.14 %) and infrastructure (19.65 %) sectors as shown in Appendix B. This trend of sector-wise performance also reflects a better practice based on the quantity of the sector's operational fugitive dust and PM emission volume. Contrastingly in Bangladesh, the reports studied failed to make a full coverage of the potential impacts and their significance on vulnerable groups including children and pregnant women [36].

The significance evaluation is the weakest stage among all IP stages, with the lowest overall score. This is echoed by other research in the UK that argues the common inferior and subpar performance of this stage is due to the lack of transparency and thoroughness [45,49]. In the Indian context [7], cast light on the superficial descriptions of the significance evaluation stage, which is evident in this paper. Also in South Africa, impact significance evaluation was unsatisfactory in 68 % of assessed reports for the oil and gas sector, which has vast negative impacts on both the environment and humans [14].

### 4.4 Performance of the mitigation measures stage

The mitigation measures stage is to resolve issues identified and found to impact the environment significantly. The mitigation commitment within the EIA report makes the EIA process a “holistic instrument” with better descriptions of environmental impacts yielding a lower impact [1]. The inclusion of mitigation measures within an EIA report is also necessary for planning permissions to be issued [55].

From the performance analysis, some of the well-performing criteria saw 85 % of projects prioritising the reduction of AQ impacts at the source. 65 % of the projects performed satisfactorily by adopting mitigation measures proposed per sector-specific EIA guidance. The description of efforts focusing on fugitive dust emissions within and surrounding the site was also satisfactory.

The lower-performing criteria in this stage included poor examination methods and a lack of clear descriptions of mitigation measures relative to the project scale. Examples include insufficient data on water requirements and dust control measures in the mining sector, as well as inadequate information on gas cleaning plants and air pollution control devices in the industrial sector. On a positive note, proposals to continuously monitor industrial and thermal power plant stacks with real-time data sent to a centralised CPCB database are given. This monitoring mechanism helps the ministry track major polluters and supports the implementation of an Emission Trading Scheme to improve transparency, predictability and cost-effective implementation, eliminating the trade-off between economic development and environmental AQ [56].

Overall, the majority of the reports lacked descriptions of calibration and maintenance plans for air monitoring and pollution control devices. The effectiveness of mitigation measures was poorly addressed if at all, and future periodic performance reviews of mitigation measures were given the least attention in all studied sectors, suggesting that Indian EIA practitioners, regulators and proponents often adopt a “build it and forget it” approach, neglecting the proper implementation and monitoring of mitigation measures. This weakness is also observed in the EIA evaluation by Ref. [57] in Bangladesh [13], in Romania and [28] in Sri Lanka.

The investigation, therefore, established that mitigation planning is the weakest in the infrastructure sector, followed by mining, industry and TPP/hydro projects (further reference in Appendix B).

### 4.5 Recommendations

The following are recommendations to improve the overall effectiveness of the two weak stages.

#### 4.5.1 Prioritisation of cumulative impact assessment within the regulatory framework

The impact prediction assessment is crucial in understanding the combined effects of multiple developments across regions [58]. In India, this practice is not effectively implemented, especially relating to AQ. The focus is often on meeting minimum national standards rather than considering the specific relief plan for the ecosystem, which undermines the effectiveness of the EIA process, reducing it to a mere administrative task.

To overcome this issue, it is important for the regulatory authority to develop a database containing data from various developments in the vicinity that is accessible by consultants conducting individual assessments for development projects. Utilising this, a comprehensive analysis of cumulative impacts can be conducted, tailoring to the environmental receptors under study. This would enhance the significance evaluation phase of the EIA process and improve its effectiveness.

#### 4.5.2 Revising the National Ambient Air Quality Standards (NAAQS)

The vast fluctuation of AQ levels for components such as PM10 and PM2.5 emissions has been reported across India since the last NAAQS guideline update in 2009 [59]. This is caused by the drastic emission changes resulting from meteorological patterns, transportation and construction. They also noted that the existing Indian NAAQS guidelines have no provision for recommended limits for AQ parameters within metropolitan and non-metropolitan jurisdictions. Furthermore, unlike most countries, India does not adopt an interim standard target setting. WHO has proposed for countries such as India, the interim emission target with project-based categorisations that can facilitate gradual improvement in AQ in a phased manner based on the locality [60]. We recommend that, rather than establishing a national recommended AQ level, the control of emissions may be set as an initial target at the state/regional level and once achieved, may look ahead to the following achievable targets in a time-bound manner. This would be realistic in improving AQ considering the varying range of particulate matter and other harmful gaseous concentrations in cities located at diverse geographical locations, as verified during various air source appropriation studies [59,61].

#### 4.5.3 Capacity building, participation and instilling accountability among all the involved stakeholders

Preparing EIA guidance documents should consider the demands of all stakeholders and specify their respective roles and responsibilities. Effective public participation in these processes reveals a vast array of social, cultural and emotional problems, so bringing them within the EIA helps make decisions more socially acceptable. To enable this, regulatory authorities' personnel and industry practitioners should collaborate in reviewing monitoring facilities more regularly and familiarise themselves with the industry's best practices. Streamlining inter-organisational collaboration would enhance information sharing. This would also aid in implementing recommendations (1) and (2) at the individual project and strategic policy, plan and programme levels [62].

#### 4.5.4 The restructuring of the EIA review mechanism by EAC and mitigation measures

The findings in this paper advocate previous research of [63], highlighting the lack of thoughtful consideration by the Expert Appraisal Committee (EAC) in their decision-making process for project proposals. The EAC failed to provide adequate justifications for mitigation recommendations and their effectiveness, leading to arbitrary decision-making. The limited time and increasing number of project proposals overwhelm the EAC members, resulting in insufficient time for thorough report review and scrutiny, adversely impacting the quality of the EIA reports. To address this, we recommend the creation of additional sector-specific committees to enhance technical expertise and reduce the workload on the EAC members.

Regarding mitigation measures, the study points out the limitations of the traditional approach of ground monitoring stations to assess changes in ambient AQ. It is deemed impractical in terms of resources, expenses and workforce. As an alternative, the use of remote sensing and GIS technologies is proposed, to utilise aerosol optical thickness in determining changes in ambient AQ across regions.

## 5 Conclusion

EIA is a comprehensive and beneficial process for a greener and just future, yet sensitive. As thoroughly discussed in this paper, EIA has gone through a lot of modifications throughout the years while being adopted in more countries. Nonetheless, numerous countries still suffer from incomplete or inefficient EIA statements. With the analysis presented in this paper, it can be seen that there is room for improvement in the IP process. Without accurate IP, the whole assessment can be adversely affected.

The findings of this study add to the existing literature in arguments of inadequacies in significance evaluation and mitigation measures as the weakest aspects of IP practice not only in India as highlighted, but also in Bangladesh, Sri Lanka and South Africa [27,28,36]. This points back to the lack of focus on defining and monitoring mitigation in different regions. From the findings and discussion outlined above, we provide several recommendations for policymakers and practitioners to improve AQ assessment and environmental outcomes.

1. **Provide clearer rationale in selecting baseline AQ sampling locations**: This should include details of the instruments used, baseline wind speed and wind direction in mining projects and ensuring an adequate number of sampling locations based on sector requirements. This method could improve the EIA process not only in India but also in Bangladesh as highlighted in the literature [36]. Furthermore, better attention should also be given to technical details of proposed projects prior to the preparation of environmental impact statements.
2. **Prioritise cumulative impact assessment within the regulatory framework**: By examining proposals in greater detail to suit specific environmental receptors, significance evaluation can be enhanced. This can include for instance full coverage of the potential impacts and their significance on vulnerable groups including children and pregnant women, where reports in Bangladesh failed to do so [36]. We also advocate for the transparency on how the analyses are conducted to remove bias in reports against significant negative impacts, as observed in a study on Canada, USA, Mexico, Brazil, UK, Australia and New Zealand [11].
3. **Revise the National Ambient Air Quality Standards (NAAQS)**: The current NAAQS, last updated in 2009, is outdated and does not reflect the fluctuating emission trends across the country. It is recommended to be revised and integrate project sector-based AQ limits that are achievable within specific timeframes under local jurisdictions, e.g. controlling the emissions as an initial target at the state/regional level. This aligns with the World Health Organisation's recommendation and has proven effective in developed countries.
4. **Enhance stakeholder accountability, participation and capacity building**: Collaborations between organisations can strengthen information sharing and transparency at both project and strategic policy levels. Measures to improve stakeholder involvement and capacity building should be implemented while simultaneously streamlining inter-organisation collaboration to include concerns of affected stakeholders, as these issues are said to be common in low and middle-income countries [13,62].
5. **Implement an improved review mechanism in the Expert Appraisal Committee (EAC) panel**: Creating sector-specific committees within the EAC can enhance the quality of report reviews by involving sector experts. This would alleviate the review load on existing EAC members.
6. **Utilise operational monitoring off-site with advanced technologies**: Adopting newer technologies using aerosol optical thickness can enable feasible and accurate monitoring of ambient AQ across regions, facilitating effective mitigation measures.

The findings in this paper provide a basis for future comparative studies on how IP practices differ in the whole of Asia or other developing regions bordering India, such as Bangladesh, Sri Lanka, and Nepal, as follow-up studies. Other potential in-depth research could be conducted to enhance the performance of impact significance evaluation and mitigation measures in the Indian EIA system. It is apparent that AQIP performance varies amongst project sectors, and sector-specific detailed reviews can be undertaken to improve the practice.

In the diversified and rapidly developing industrial nation, conserving the natural environment in its natural condition is becoming more difficult. Implementing efficient EIA procedures can add to or lessen the severity of this problem's consequences. Therefore, there is significant potential for research to examine the EIA practice to verify its efficacy and give recommendations for improvement. This study has contributed to furthering sustainable development for our shared future.

## 6 Limitations and future research

While it is acknowledged that including a larger and more diverse sample of EIA reports would have strengthened the study's findings, the sample size of 20 reports aligns with previous studies (e.g. Refs. [13,27]). Practical limitations, such as restricted research time and data analysis constraints, influenced the sample size selection. During the assessment process, some reports were found to be unsuitable for the study and were revised. The reasons for inapplicability included obscured pages, including maps with sampling sites, and in some cases, the absence of the AQ chapter altogether.

There are limitations related to the review package criteria used in this study, as evaluating technically complex EIA reports can be subjective, despite being based on scientific foundations. To address this, trained experts are typically involved in the evaluation process to minimise bias [64]. EIA reports undergo a double review by two individuals to mitigate subjectivity. However, this process was not feasible in this study. Nonetheless, a critical and questioning approach was maintained during the evaluation process.

This article has provided insights into the quality of EIA reports in India, which could spark interest for research to compare with other developing economies bordering India, such as Bangladesh, Sri Lanka, Bhutan, Nepal, Pakistan and China. Other potential in-depth research could be conducted to enhance the performance of impact significance evaluation and mitigation measures in EIA.

## Data availability statement

Data supporting this study are included within the article.

## CRediT authorship contribution statement

**Hairul Sharani Mohd Radzuan:** Writing – review & editing, Supervision, Project administration, Formal analysis. **Jeff Martin:** Writing – original draft, Visualization, Methodology, Formal analysis, Data curation, Conceptualization.

## Declaration of competing interest

The authors declare that they have no known competing financial interests or personal relationships that could have appeared to influence the work reported in this paper.

## Figures and Tables

**Table:** 

No	Title	Published year
**Mining (Coal and Non-Coal)**		
1	Expansion in Coal Production Capacity from 1.20 MTPA to 3.00 MTPA (Normative) and 3.75 MTPA (Peak) & Expansion in Land Area from 479.16 ha to 706.28 ha in Majri Area, Maharashtra: Prepared by Central Mine Planning and Design Institute Limited.	2020
2	Expansion in Coal Production Capacity from 2.00 MTPA to 3.50 MTPA with 614.69 ha area in Phase-I Umrer Area, Nagpur District, Maharashtra: Prepared by Central Mine Planning and Design Institute Limited.	2020
3	Expansion in Limestone mining from 1.6 MTPA to 2.4 MTPA in Mangrol-Tilakhera region, Chittorgarh District, Rajasthan: Prepared by Enkay Enviro Services Pvt Ltd.	2020
4	Kumaraswamy Iron Ore Mine 7.0 MTPA ROM Iron Ore production capacity of NMDC Limited, Tehsil Sandur, Ballari District, Karnataka: Prepared by Ecomen Laboratory Pvt Ltd.	2019
5	Sugala Limestone and Marl Mine with 1.29 MTPA production capacity at Kodinar, Gujarat: Prepared by JM Environet Pvt Ltd.	2019
**Infrastructure (Building and Construction/Township and area development/Airports)**		
1	Proposed setting up of common incinerator of 10 TPD, common biomedical waste treatment facility of 10 TPD and 5 MLD common effluent treatment plant at KIADB Harohalli Industrial Area, Ramanagar District, Karnataka: Prepared by Mother Earth Environ Tech Pvt Ltd.	2021
2	Development of KINFRA Petrochemical Park at Thiruvankulam village, Ernakulam District, Kerala: Prepared by Voyants Solutions Private Limited.	2020
3	Deepening and widening of approach channel to second chemical berth at Pir Pau, Mumbai: Prepared by Enkay Enviro Services Pvt Ltd.	2021
4	Development of economic corridors, inter-corridors and coastal roads of 240.372 km length towards efficient freight movement in state of Odisha: Prepared by CEMC and EIS Pvt Ltd.	2021
5	Development of greenfield Jewar International Airport Phase 1 and 2, Uttar Pradesh: Prepared by Greencindia consulting Pvt Ltd.	2019
**Manufacturing Industries**		
1	Expansion of Chaliyama Steel plant (Steel Production: 1.0395 to 2.8805 MTPA, Area: 239.64 Ha to 377.75 Ha): Prepared by Min Mec Consultancy Pvt Ltd and CEMC Pvt Ltd.	2020
2	Development of Integrated Cement plant with Captive powerplant, WHRS, DG sets and residential colony at Gothra, Jhunjhunu, Rajasthan: Prepared by J.M Environet Pvt Ltd.	2020
3	Development of associated project facilities for production of BS VI auto fuel at MRPL, Mangalore, Karnataka: Prepared by CSIR-NEERI.	2017
4	Proposed production of technical grade pesticides and intermediates, Total Production capacity 1099 MT/month in Vagra, Bharuch, Gujarat: Prepared by Anand Environmental consultants Pvt Ltd.	2020
5	Proposed expansion of Distillery from 120 KLPD to 300 KLPD Ethanol with Captive power plant expansion from 5 MW to 8 MW in Bagalkot, Karnataka: Prepared by Samrakshan.	2020
**Thermal and Hydro Power projects**		
1	Development of 500 MW coal based captive thermal power plant at Hathneora, Chhatisgarh: Prepared by EQMS India Pvt Ltd.	2015
2	Development of 3 × 800 MW Talabira Thermal Power plants in villages of Jharsuguda and Sambalpur District, Odisha: Prepared by ABC Techno Labs India Pvt Ltd.	2020
3	Proposal for 14.5 MW Coal Captive cogeneration powerplant at Puthukkadu, Erode, Tamil Nadu (proposed site in close proximity to a tiger reserve eco-sensitive zone, hence falling under Category A): Prepared by Cholamandalam MS Risk services Ltd.	2021
4	Development of Rammam Hydropower project 3 × 40 MW in Darjeeling and West Sikkim, West Bengal and Sikkim: Prepared by SV Enviro labs and consultants.	2021
5	Development of Lakhwar Hydro project (300 MW) in Dehradun, Uttarakhand: Prepared by RSET Pvt Ltd.	2020

**Table:**